# Relationship between testosterone and male bladder cancer

**DOI:** 10.1038/s41598-023-34646-2

**Published:** 2023-08-08

**Authors:** Wei Tan, Liang Gao, Ye Yuan, Hao Huang, Yadong Li, Yuanqing Gou, Zili Hu

**Affiliations:** 1 Department of Urology, Chongqing Traditional Chinese Medicine Hospital, Chongqing, China; 2 Department of Urology, The Second Affiliated Hospital of Chongqing Medical University, Chongqing, China

**Keywords:** Cancer screening, Tumour biomarkers, Urological cancer

## Abstract

Researches had proven that the occurrence of bladder cancer (BC) is much higher in men than those in women, which induced us to explore whether androgen plays a role in BC. A total of 147 patients who were diagnosed with primary BC by histopathological biopsy were included. Meanwhile 154 non-tumor patients were matched as the control group. The continuous variables were expressed as median (interquartile range, IQR) and compared by Mann–Whitney U test, for the reason that the data were not matched the requirementsthe of normal test. A Chi-square test was used to compare the categorical variables, which were expressed as frequency (percentage). Meanwhile univariate and multivariate logistic regression was done to further evaluating the potential independent factor of BC. *P* < 0.05 was considered statistically significant. Univariate multivariate analyse showed significant difference between two groups in hemoglobin (OR 0.979, 95% CI 0.968–0.991, *P* < 0.001), hypertension (OR 3.026, 95% CI 1.731–5.288, *P* < 0.001), diabetes (OR 4.294, 95% CI 1.887–9.771, *P* = 0.001) and smoking (OR 1.729, 95% CI 1.096–2.729, *P* = 0.019). Furthermore, multivariate logistic regression analysis was conducted to eliminate the interference of confounding factors, which showed that testosterone seems to be great correlated with the BC (OR 1.002, 95% CI 1.000–1.003, *P* = 0.017). Similar results were also found in hemoglobin (OR 0.981, 95% CI 0.968–0.993, *P* = 0.002), hypertension (OR 2.780, 95% CI 1.509–5.120, *P* = 0.001), diabetes (OR 3.313 95% CI 1.373–7.991, *P* = 0.008) and smoking (OR 1.938, 95% CI 1.184–3.174, *P* = 0.009). As a conclusion, our study showed that there was significant correlation between serum total testosterone levels and the occurrence of BC, similar results were shown in hemoglobin, hypertension, diabetes and smoking.

## Introduction

Bladder cancer (BC), is one of the most frequent malignancies in urinary system^1^. Data from the Global Cancer Observatory (GCO, https://gco.iarc.fr/) showed that, 573,278 individuals were diagnosed with BCa and 212,536 individuals died of BCa in 2020, making BCa the tenth most common tumor worldwide^1^. Meanwhile, it has been proven that the occurrence of BC is about three to five times higher in men than those in women, which induce us to explore that whether androgen has played a role in BC^2^.

Testosterone is the predominant endogenous androgen in human bloodstream. Previous studies had proved that testosterone plays an important role in the human system, especially in cardiovascular diseases and prostate cancer. Lorigo found that changes of testosterone expression level can infulence the incidence rate of cardiovascular diseases by regulating metabolic syndrome, type 2 diabetes mellitus, obesity, atherosclerosis, dyslipidaemia and hypertension^3,4^. Meanwhile, androgen deprivation therapy had became the basis of the prostate cancer treatment. Nevertheless, few studies focused on the relationship between testosterone and bladder, especially in bladder cancer. Lorenzetti found that low testosterone levels can induce apoptosis by acting 3-caspase dependent signaling in the bladder wall of male rats^5^. Nakazawa found that the expression of angiotensin II receptor mRNA and caspase-3 protein significantly increased in the rat bladder after castration and can be reversed by testosterone administration^6^. Meanwhile, McBeth reported that the expression of androgen receptor (AR) in BC tissue ranges from 17 to 78%^7^. However, existing clinical studies have also showed conflicting results. The study of Sanguedolce showed that the lack of AR expression was significantly correlated to recurrence of non-muscle-invasive bladder cancer^8^. Kafkasli revealed that testosterone level was not found to be associated with any of the categories of bladder tumor aggressiveness^9^. Therefore, we performed this study to further explore whether there was a correlation between serum total testosterone levels and BC.

## Materials and method

This study was approved by the Institutional Review Board of the Second Affiliated Hospital of Chongqing Medical University and in accordance with the Helsinki Declaration. Meanwhile, all the informed consent was obtained from all subjects and/or their legal guardians.

We retrospectively included 147 male bladder cancer patients who were diagnosed with primary BC by histopathological biopsy at the Second Affiliated Hospital of Chongqing Medical University from January 2007 to April 2022. Meanwhile, 154 non-tumor patients were matched as the control group. Baseline information was shown in Table 1. All participants in the study were male. Blood samples were taken between 06:00 and 08:00 a.m. after fasting overnight.

**Table 1:** Baseline.

Characteristics	BC	Control	Z/χ^2^	*P*
Number	147	154		
Age (year)	69 (63, 77)	70 (62, 76)	− 0.619	0.536
Testosterone (ng/dL)	353 (232, 486)	332 (248, 449)	− 0.908	0.364
Lymphomonocyte (10^9/L)	1.3 (0.99, 1.64)	1.21 (0.94, 1.63)	− 0.870	0.384
hemoglobin (g/L)	131 (115, 142)	136 (127, 148)	− 3.076	0.002
Hypertension	51 (34.7%)	23 (14.9%)	15.837	< 0.001
Diabetes	28 (19%)	8 (5.2%)	13.707	< 0.001
CHD	13 (8.8%)	8 (5.2%)	1.543	0.214
Smoking	83 (56.5%)	66 (42.9%)	5.569	0.018
Drinking	58 (39.5%)	45 (29.2%)	3.500	0.061
Grade				
Low	83 (56.5%)	NA		
High	64 (43.5%)	NA		

CHD, Coronary atherosclerotic heart disease; NA, not applicable.

All patients included in the study had no history of malignant tumors, including bladder cancer. Patients who had received any kind of androgen inhibition or supplementary treatment, or had testicular loss or atrophy caused by any reasons were excluded from the study. Meanwhile, patients who did not receive testosterone test in our hospital were also excluded.

Data of age, living habits (including smoking, and drinking), basic diseases (including hypertension, diabetes, and coronary atherosclerotic heart disease (CHD)), serum testosterone levels, some other serum indicators (including lymphomonocyte and hemoglobin), and pathological results of high- and low-grade were included.

We used the SPSS25.0 statistical software package for statistical analysis. The continuous variables were expressed as median (interquartile range, IQR) and compared by Mann–Whitney U test, for the reason that the data were not matched the requirementsthe of normal test. A Chi-square test was used to compare the categorical variables, which were expressed as frequency (percentage). Meanwhile univariate and multivariate logistic regression was done to further evaluating the potential independent factor of BC. *P* < 0.05 was considered statistically significant.

### Ethical compliance

All procedures performed in studies involving human participants were in accordance with the ethical standards of the institutional and/or national research committee and with the 1964 Helsinki Declaration and its later amendments or comparable ethical standards.

## Results

### Characteristics of included patients

A total of 301 patients were enrolled into our study, including 147 BC male patients and 154 non-tumor male patients. Mann–Whitney U test was taken to compare the data between the BC group and non-tumor group because of non-normal distribution. Results were shown in the Table 1. Significant differences were shown in hemoglobin, hypertension, diabetes and smoking (*p* < 0.05).

### Univariate and multivariate analyses

Univariate and multivariate analyses of each parameter are summarized in Table 2. Univariate multivariate analyse showed significant difference between two groups in hemoglobin (OR 0.979, 95% CI 0.968–0.991, *P* < 0.001), hypertension (OR 3.026, 95% CI 1.731–5.288, *P* < 0.001), diabetes (OR 4.294, 95% CI 1.887–9.771, *P* = 0.001) and smoking (OR 1.729, 95% CI 1.096–2.729, *P* = 0.019). Meanwhile no significant differences were shown in age (OR 1.009, 95% CI 0.985–1.033, *P* = 0.456), testosterone (OR 1.000, 95% CI 0.999–1.002, *P* = 0.564), lymphomonocyte (OR 1.135, 95% CI 0.772–1.670, *P* = 0.520), CHD (OR 1.771, 95% CI 0.712–4.405, *P* = 0.219), and drinking (OR 1.579, 95% CI 0.977–2.550, *P* = 0.062).

**Table 2:** Univariate and multivariate analysis of bladder cancer patients and non tumor patients.

Characteristics	Univariate			Multivariate		
	OR	95% CI	*P*	OR	95% CI	*P*
Age (year)	1.009	(0.985, 1.033)	0.456			
Testosterone (ng/dL)	1.000	(0.999, 1.002)	0.564	1.002	(1.000, 1.003)	0.017
Lymphomonocyte (10^9/L)	1.135	(0.772, 1.670)	0.520			
Hemoglobin (g/L)	0.979	(0.968, 0.991)	< 0.001	0.981	(0.968, 0.993)	0.002
Hypertension	3.026	(1.731, 5.288)	< 0.001	2.780	(1.509, 5.120)	0.001
Diabetes	4.294	(1.887, 9.771)	0.001	3.313	(1.373, 7.991)	0.008
CHD	1.771	(0.712, 4.405)	0.219			
Smoking	1.729	(1.096, 2.729)	0.019	1.938	(1.184, 3.174)	0.009
Drinking	1.579	(0.977, 2.550)	0.062			

CHD, Coronary atherosclerotic heart disease; OR, odds ratio; 95% CI, 95% confidence interval.

Subsequently, we conducted multivariate logistic regression analysis to eliminate the interference of confounding factors. As is shown in Table 2, testosterone seems to be great correlated with the BC (OR 1.002, 95% CI 1.000–1.003, *P* = 0.017). Similar results were also found in hemoglobin (OR 0.981, 95% CI 0.968–0.993, *P* = 0.002), hypertension (OR 2.780, 95% CI 1.509–5.120, *P* = 0.001), diabetes (OR 3.313 95% CI 1.373–7.991, *P* = 0.008) and smoking (OR 1.938, 95% CI 1.184–3.174, *P* = 0.009).

## Discussion

In our study, we found that testosterone is an independent factor of BC after adjustment for potential confounding factors.

BC is one of the most common malignant tumors with great recurrence rate, even though early endoscopic resection have been carried out, which made patients undertake great physical mental and economic pressure. Therefore, to explore the risk factors of bladder cancer and reduce the incidence rate and recurrence rate is the urgent need of human economy and health need.

Previous research found that the incidence of BC is related to androgen receptor and testosterone. Izumi et al. found that androgen-mediated AR signals promote bladder carcinogenesis by down-regulating the expression of UDP-glucuronosyltransferases in the bladder^10^. Positive AR expression in non-muscle invasive bladder cancer (NMIBC) was an independent factor predicting lower risk of multiple recurrences^11^. Meanwhile, some researchers found that some AR-targeted drugs could be useful in the treatment of BC. Tyagi et al. found that the combination of enzalutamide and cisplatin synergistically inhibited the growth of BCa cells more efficiently than a single agent, revealing that AR antagonists might be a possible choice in the treatment of BC^12^. Imada et al. found that male rats been treated by androgen deprivation or 5α-reductase inhibitor might have a lower risk of BC^13^. On the basis of some similar studies, some researchers began to investigate whether treatment with a 5α-reductase inhibitor or androgen deprivation could reduce the incidence or recurrence of BC in male patients, which revealed some positive results^14–17^.

Furthermore, androgen deprivation also showed effect on the recurrence of BC. In a retrospective, single-institution study, androgen suppression therapy was found to be associated with a lower risk of recurrence in NMIBC^16^. Izumi K et al. also found that patients with prostate cancer who had received androgen deprivation therapy (ADT) showed a significantly lower risk of BC recurrence and also had a significantly smaller number of recurrence episodes, compared to the control patients^18^.

However, different results were also reported. Wallner et al. carried out a study focusing on those men with localized prostate cancer, which revealed that ADT was not associated with an increased risk of second primary BC^19^. Meanwhile another study also found that there was an increased risk of second primary BC among men treated with ADT in PCa patients compared to the general population^20^. Therefore, in order to further clarify the relationship between testosterone and bladder cancer, we conducted this retrospective study.

In addition to testosterone, we also showed that hemoglobin may be the possible correlated factor to BC. Just as the previous studies showed, a retrospective study conducted by Zhang showed that hemoglobin is an independent risk factor affecting the prognosis of patients with MIBC^21^. Similarly, Celik found that preoperative anemia status of BC patients is associated with decreased OS, but not with cancer-specific survival^22^. Chen revealed that BC patients with anemic are likely to have a shorter recurrence-free survival (RFS) and OS than non-anemic patients^23^. And similar result was also found in our study.

As a result, our study showed that testosterone is an independent factor to BC, so does the hemoglobin, hypertension, diabetes and smoking. Howbeit, several limitations should also be taken into consideration in our study. Firstly, our study is not a prospective study, which would affect the quality of results. Secondly, our study was a single-center study, the sample size was small. More importantly, because of the deficiency of follow-up, we could not provide more information on the relationship between serum total testosterone and the recurrence or survival of BC patients.

## Conclusion

Our study showed that there was significant correlation between serum total testosterone levels and the occurrence of BC, similar results were shown in hemoglobin, hypertension, diabetes and smoking.

## Data Availability

All data generated or analysed during this study are included in this published article.
